# A radiographic, deep transfer learning framework, adapted to estimate lung opacities from chest x-rays

**DOI:** 10.1186/s42234-022-00103-0

**Published:** 2023-01-03

**Authors:** Avantika Vardhan, Alex Makhnevich, Pravan Omprakash, David Hirschorn, Matthew Barish, Stuart L. Cohen, Theodoros P. Zanos

**Affiliations:** 1 Institute of Health System Science, Feinstein Institutes for Medical Research, Northwell Health, Manhasset, NY 11030 USA; 2 Institute of Bioelectronic Medicine, Feinstein Institutes for Medical Research, Northwell Health, Manhasset, NY 11030 USA; 3 Donald and Barbara Zucker School of Medicine at Hofstra/Northwell, Northwell Health, Hempstead, NY 11549 USA; 4 Department of Information Services, Northwell Health, New Hyde Park, NY 11042 USA

**Keywords:** Chest X-ray (CXR), Deep transfer learning, Heatmap concordance, Lung opacity, Ordinal classification, Pretrained model

## Abstract

Chest radiographs (CXRs) are the most widely available radiographic imaging modality used to detect respiratory diseases that result in lung opacities. CXR reports often use non-standardized language that result in subjective, qualitative, and non-reproducible opacity estimates. Our goal was to develop a robust deep transfer learning framework and adapt it to estimate the degree of lung opacity from CXRs. Following CXR data selection based on exclusion criteria, segmentation schemes were used for ROI (Region Of Interest) extraction, and all combinations of segmentation, data balancing, and classification methods were tested to pick the top performing models. Multifold cross validation was used to determine the best model from the initial selected top models, based on appropriate performance metrics, as well as a novel Macro-Averaged Heatmap Concordance Score (MA HCS). Performance of the best model is compared against that of expert physician annotators, and heatmaps were produced. Finally, model performance sensitivity analysis across patient populations of interest was performed. The proposed framework was adapted to the specific use case of estimation of degree of CXR lung opacity using ordinal multiclass classification. Acquired between March 24, 2020, and May 22, 2020, 38,365 prospectively annotated CXRs from 17,418 patients were used. We tested three neural network architectures (ResNet-50, VGG-16, and ChexNet), three segmentation schemes (no segmentation, lung segmentation, and lateral segmentation based on spine detection), and three data balancing strategies (undersampling, double-stage sampling, and synthetic minority oversampling) using 38,079 CXR images for training, and validation with 286 images as the out-of-the-box dataset that underwent expert radiologist adjudication. Based on the results of these experiments, the ResNet-50 model with undersampling and no ROI segmentation is recommended for lung opacity classification, based on optimal values for the MAE metric and HCS (Heatmap Concordance Score). The degree of agreement between the opacity scores predicted by this model with respect to the two sets of radiologist scores (OR or Original Reader and OOBTR or Out Of Box Reader) in terms of performance metrics is superior to the inter-radiologist opacity score agreement.

## Introduction

Numerous disease processes affecting the lungs can result in lung opacities that are visualized on various imaging modalities. Chest radiographs (CXRs) are the most ubiquitous imaging tests for diagnosing respiratory diseases worldwide (Mettler Jr. et al., 2009; United Nations Scientific Committee on the Effects of Atomic Radiation, 2008). Importantly, the degree of lung opacities on CXRs have been shown to predict patient outcomes (Au-Yong et al., 2022; Balbi et al., 2021; Voigt et al., 2022). Clinically, CXR reports are typically not standardized, and the language used to convey certainty of a diagnosis is highly variable (Makhnevich et al., 2019; Makhnevich et al., 2022). Further, degree of lung opacity is typically estimated visually and oftentimes reported using vague, nonstandard language (i.e., mild, hazy, severe, extensive, patchy) (Little, 2022). Quantitative or semi-quantitative scores have been developed to determine degree of lung opacity, but because they vary in reproducibility and are time consuming to quantify, they are rarely used in clinical practice (Au-Yong et al., 2022; Monaco et al., 2020; Reeves et al., 2021).

The established infrastructure for CXR availability, worldwide, represents a unique opportunity to leverage current machine learning (ML) models to improve the diagnostic and prognostic capabilities of a relatively inexpensive diagnostic test. The benefit of using ML models in medical image processing is evident by improvements in the diagnostic speed, accuracy, and reproducibility for detecting and quantifying numerous pathologic findings as compared to radiologists (Erickson et al., 2017; Rajpurkar et al., 2017; Seah et al., 2021; Wang et al., 2021). For interpretation of CXRs specifically, application of deep learning could help overcome human error due to fatigue and other interruptions (Brady, 2017). In addition, deep learning systems trained on sufficiently large datasets can assist and augment radiologists given the often-small number of experienced radiologists tasked with reading large volumes of CXRs taken every day (Wu et al., 2020). For instance, in a large-scale, retrospective, multireader, multicase study, in 80% of the study CXRs, the accuracy of radiologists assisted by a deep learning model was superior to that of unassisted radiologists and non-inferior for 95% of findings (Seah et al., 2021). Recently, the unpredictability of patient outcomes during the coronavirus disease 2019 (COVID-19) pandemic required many clinicians to use CXRs - not only as a diagnostic but also a prognostic tool. By applying computer vision and deep learning on CXRs, multiple diagnostic and prognostic tools were proposed, including models predicting the length of hospital stays, mortality and ventilation, ICU admission, and likelihood of conversion to severe infection (Au-Yong et al., 2022; Khan et al., 2020; Mushtaq et al., 2021; Roberts et al., 2021).

Despite the advancement in CXR image processing via deep learning based on Convolutional Neural Networks (CNNs), the diagnostic and prognostic capabilities of CXRs in the field of pneumonia research are still limited by many factors. Many ML models have problems with robustness, accuracy, and reliability due to low number of images used, imbalanced and biased datasets, black-box approaches failing to ensure that models do not overfit or use non-clinical features, and incomplete comparisons due to code availability, differences in processing pipelines, data balancing schemes, and validation procedures (Roberts et al., 2021). In a review study of image-based COVID-19 diagnostic models, only 7 out of 32 papers used a dataset of over 2000 images. Studies using a limited number of images to train such models can lead to low classification accuracy, high bias, and limited generalization of the developed models (Oh et al., 2020). Moreover, (Alghamdi et al., 2021; Roberts et al., 2021), and the few models that identify the degree of opacity employ an often low number of data points (< 300) and lack testing of multiple architectures and preprocessing schemes (Cohen et al., 2020; Li et al., 2020). Finally, when heatmaps are employed to provide insights into how the model generates predictions, one observes a lack of a unifying metric that quantifies the clinical relevance of image regions that the model considers salient, making comparisons to other models based on the heatmap qualitative (Samek et al., 2016).

The purpose of this study is to develop a robust medical imaging deep learning framework and adapt it to estimate CXR lung opacity. To this end, we utilized prospectively quantified lung disease burden by radiologists at the time of dictation on 38,365 CXRs. We used this large dataset to test a set of models trained using all combinations of three different data balancing schemes, three different ROI (Region of Interest) segmentation strategies, and three different pre-trained CNN architectures to perform ordinal classification. Further, a transfer learning strategy that employed fine-tuning on a number of CNN layers (in addition to the commonly implemented fine-tuning on the fully connected network) was used. The output of these models was a lung-specific (i.e., left or right lung) opacity score, and a robust multi-level approach was used to determine the best model. We evaluated the models via testing on cross-validated data folds and out-of-the-box datasets, using ordinal specific performance metrics such as the macro-averaged mean absolute error (macro-averaged MAE), and proposed a novel metric of network heatmap concordance to evaluate the clinical relevance of model-generated salient features. We identified the best performing models, performed robustness analysis, evaluated performance metrics across multiple demographic groups, produced visualizations using heatmaps, and showcased the benefits of this approach.

## Methods

A multi-level pipeline is proposed for image classification based on the core principle of transfer learning with fine tuning. After the initial stage of data preparation, where images are converted, included, or excluded in the study based on specific criteria and preprocessed accordingly, images are analyzed using a combinatorial approach of different ROI extraction/segmentation approaches, different sampling/data balancing methods, and different neural network architectures. Once all combinations of these three modeling components are trained and tested, a list of the top performing models based on the metric of choice is constructed and these models then undergo subsequent robustness analysis where they are tested across multiple data folds using K-fold cross validation. Their heatmaps are also assessed for concordance with clinical relevance using a quantitative ROI overlap score. Using these metrics, the best model is chosen and is finally compared to expert radiologist annotations in an out-of-the-box dataset. Performance analysis is also performed on the chosen model across multiple population groups based on race, sex, and COVID-19 status, and subgroup performance is reported. The framework is designed to optimize model selection while also combining computational efficacy and robustness.

As a use case of this pipeline, we analyzed 38,365 CXRs to estimate lung disease burden. In the first level, every combination of the following were implemented, resulting in a total of 27 models (each for left and right lung opacity detection – 54 models in total): (a) three image segmentation schemes for ROI generation (i.e., lung segmentation, spine segmentation and no segmentation), (b) three data-balancing schemes for creation of balanced training data for transfer learning (i.e., random under-sampling, oversampling and double-stage sampling), and (c) three network architectures (i.e., VGG-16, ResNet-50 and CheXNet-121. Performance is reported on all above models that were created, based on multiple metrics).

### Study design, setting, and population

This retrospective cohort study included consecutive adult patients, aged 18 and older, while hospitalized in 1 of 12 acute care hospitals across a multihospital integrated healthcare network in the New York metropolitan region between March 24, 2020, and May 22, 2020. During the time of the study at our institution, all emergency department (ED) patients and inpatients (IP) with single-view CXR had their CXRs prospectively quantified at the time of dictation by board certified radiologists of varied experience, referred to as the OR (Original Reader) in subsequent sections. The study was performed with institutional review board (IRB # 19–0596 and 20–0781) approval and waiver of informed consent. All study data was obtained from the Radiology Information System (RIS) and the enterprise inpatient Electronic Health Record (EHR; Sunrise Clinical Manager, Allscripts, Chicago, IL).

### CXR imaging quantification by radiologist

Lung disease burden was prospectively quantified by radiologists at the time of dictation with each lung annotated by the degree of lung opacity as clear (0%), mild (1–33%), moderate (34–66%), or severe (67–100%). This was performed using discrete fields in the radiology reporting software (Nuance Powerscribe) using a pop-up upon a radiologist finalizing a CXR report with results stored in a secure radiology database. If the radiologist reported lung opacity in the report using the reporting system template, the data was stored in the radiology database without the use of a pop-up. This score is referred to as the OR or Original Reader score in later sections of the paper.

### Patient variables

Age was obtained as a continuous variable and sex as a binomial variable. Patient status was noted at the time of imaging as inpatient (IP), outpatient (OP), or Emergency Department (ED). Race was categorized as White, Black, Asian, other, or unknown. Diagnosis of COVID-19 for each CXR was confirmed by a positive result on at least one Polymerase Chain Reaction (PCR) test, either before the date that the CXR was taken or within 7 days after the CXR was taken. A patient was categorized as COVID-19 positive if they had at least a single positive PCR test during hospitalization.

### Classification, balancing, evaluation

**Fig. 1:**
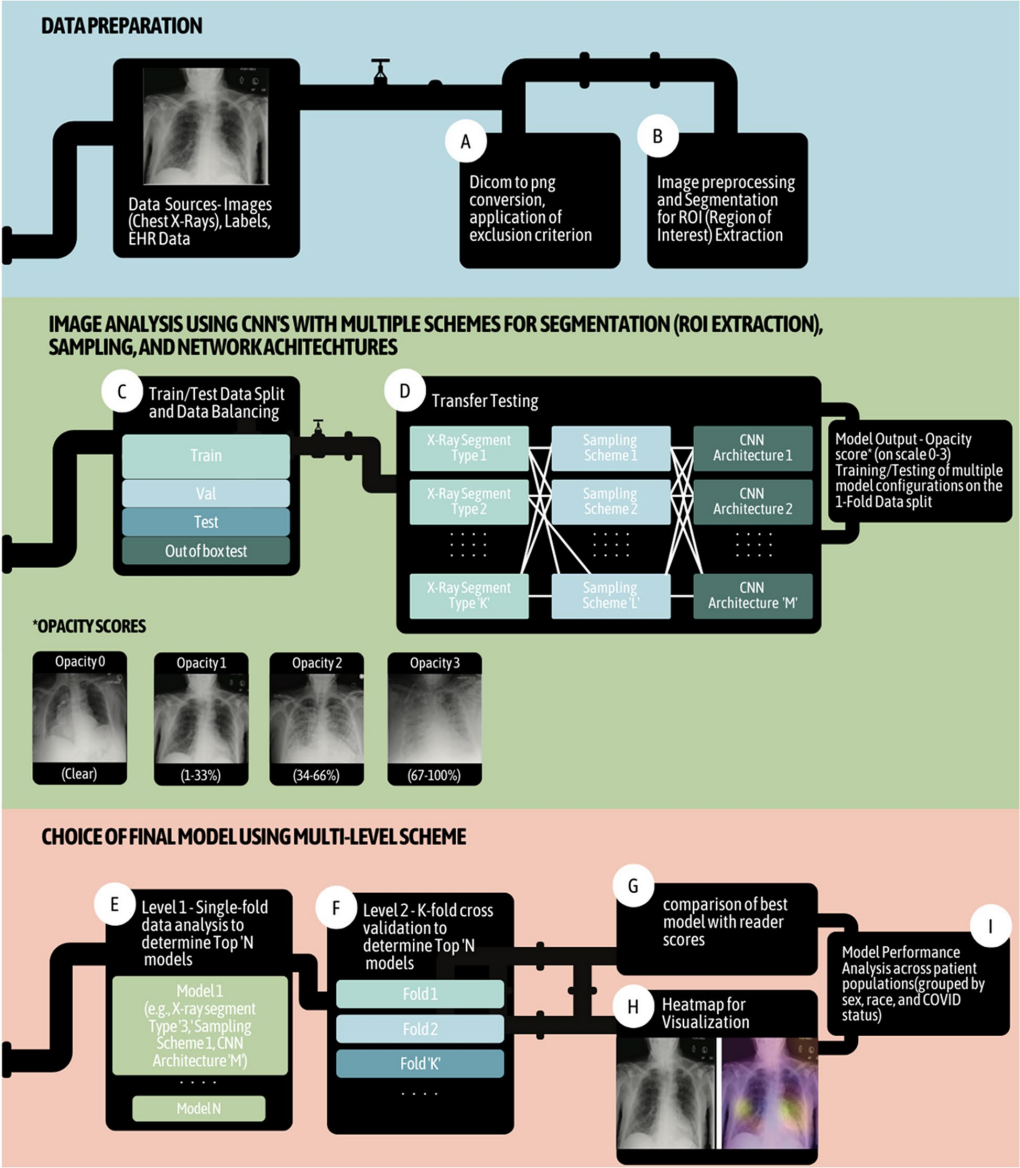
Schematic of the proposed pipeline. The different steps of the pipeline are denoted with letter from A–I. Overall pipeline of CXR framework for scoring opacity using deep learning. Steps include (**A**) Data Preparation: DICOM to PNG conversion and application of exclusion criteria; (**B**) image preprocessing and ROI extraction; (**C**) train/test data split and data balancing; (**D**) transfer learning setup for testing models generated using multiple combinations of X-ray segmentation schemes, sampling schemes to overcome dataset bias, and CNN architectures; (**E**) level 1 – single-fold analysis to determine top ‘N’ models; (**F**) level 2 – K-fold cross validation to determine best model; (**G**) comparison of best model with reader scores; (**H**) heatmap for visualization; and (**I**) model performance analysis across different patient populations, grouped by sex, race, and COVID-19 status

Multiple models with all possible combinations of network architectures, data balancing, and image segmentation schemes were tested (Fig. 1), and the best model was determined using a series of steps detailed in Section 2.4.5. The opacity predicted by the best model was evaluated using an Out Of Box test set (OOB test set) and performance was compared with scores given by expert radiologists.

#### Step A: DICOM to PNG conversion and application of exclusion criteria

The initial set of 83,197 DICOM images (20,180 patients) were processed using the following steps. When multiple scans were obtained for the same patient with the same accession number, only the first image was retained, and the others (often post-processed versions of the original X-ray via filtering) were discarded (38,575 images and no patients were discarded). In addition, the following types of images were also discarded: document-type images (< 1% of total) that were mistakenly included as X-rays (112 images, 87 patients), and images with read errors/acquisition issues (1580 patients, 4318 images). Images with read errors/acquisition issues that were discarded were identified based on the following criteria – images with a single accession number but multiple acquisition times, X-rays with missing content time, and images that could not be read or saved for various reasons.

**Fig. 2:**
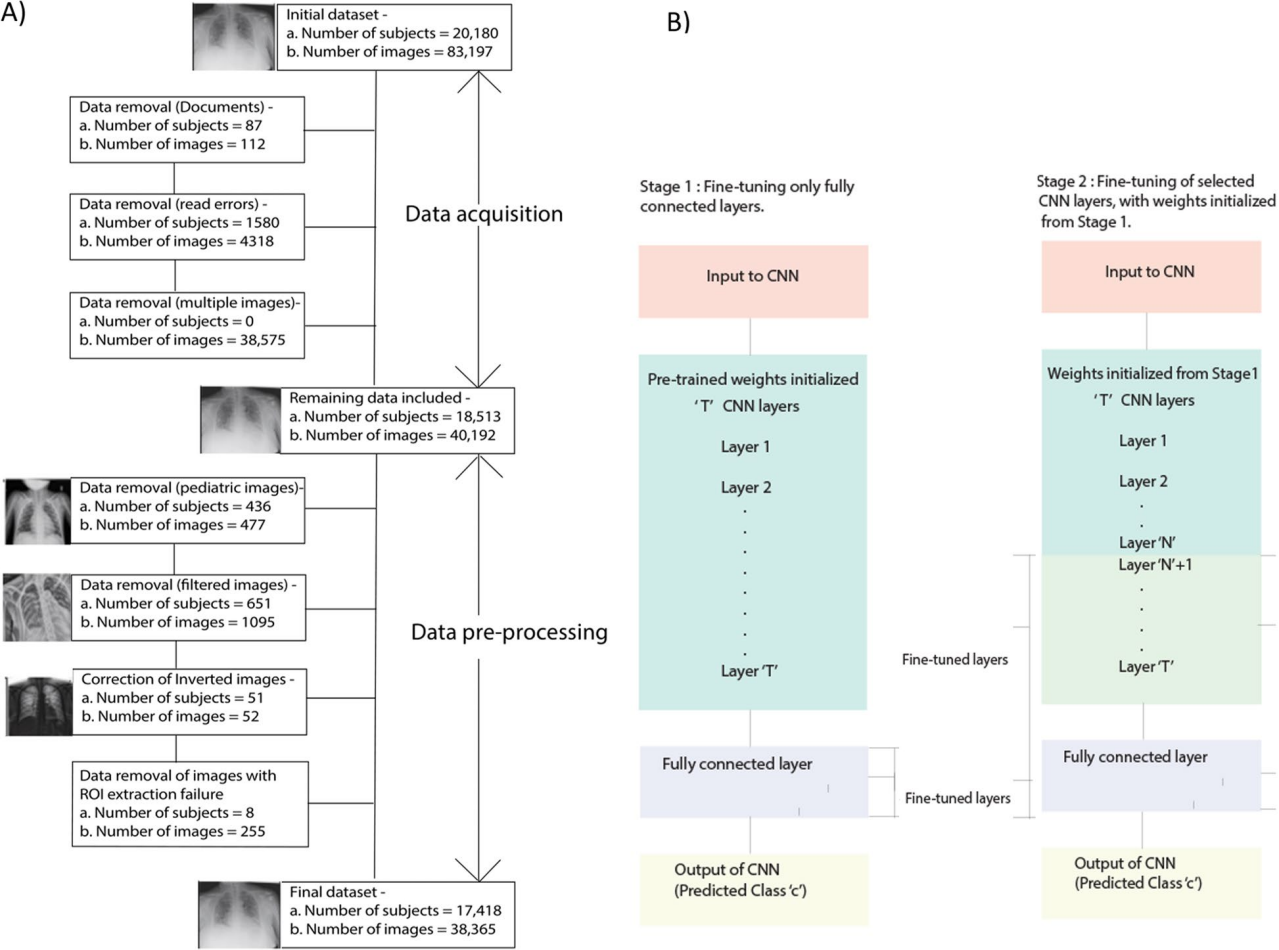
Schematic of exclusion criteria and transfer learning framework. (**A**) Data exclusion criteria: multiple stages of image acquisition, DICOM to PNG conversion, and application of other exclusion criteria during the data pre-processing stage prior to final data creation; and (**B**) transfer learning framework used to leverage weights from pre-trained models along with our dataset of CXRs with scored opacities. The two different schemes and stages explored using this framework are portrayed

Following the preparation of data, additional criteria were applied to obtain the final set of images to pass through the deep learning framework (Fig. 2). The images remaining from the initial DICOM image post-processing as previously described were then passed through rejection criteria. Of these, the following images were discarded: (a) images belonging to pediatric subjects under 18 years of age (436 patients, 477 images), (b) images that were not the original X-ray but post-processed images using a filter (651 patients, 1095 images) (these were detected using a customized pre-trained ResNet-50 model based on transfer learning). Images that were CXR negatives were also detected using a second customized pre-trained ResNet-50 model (51 patients, 52 images), and the pixel intensities of these images were transformed to make them match the regular X-ray. Further, in the image segmentation pipeline described in the next section, 255 CXRs belonging to 8 patients were discarded due to errors in lung ROI segmentation. Following these data exclusion steps, the remaining images (38,365 CXRs) were further processed in the next steps of the pipeline.

#### Step B: Image preprocessing and segmentation for ROI extraction

Prior to using deep learning–based solutions to classify extent of opacity, preprocessing lung CXR images via masking of non-clinical area can minimize the presence of irrelevant features (Rajaraman et al., 2020). Further, since the goal of the algorithm presented here is to classify left lung and right lung opacities separately, independent left and right lung specific algorithms were developed for predicting each of these opacities. Said lung algorithms could potentially benefit from input images that are specific to the lung side under study. Toward this goal of independent left/right algorithm development, we tested multiple image preprocessing schemes prior to passing the images through the deep learning framework: (a) no segmentation scheme (using the complete CXR image with no removal of regions), (b) lung segmentation scheme (extracting only the specific lung ROI [left lung or right lung] using U-Net based semantic segmentation), and (c) spine segmentation scheme (division of the CXR image into left lung and right lung ROIs based on U-Net segmentation of the spine) (Ronneberger et al., 2015).

For both lung and spine-based ROI extraction, a standard U-Net semantic segmentation was used. The Japanese Society of Radiological Technology (JSRT) dataset was used to obtain 247 training CXR images with pixel resolution 256 × 256 (Shiraishi et al., 2000). For lung segmentation, the existing lung masks for said JSRT images were used (van Ginneken et al., 2006). For spine segmentation, masks on the JSRT dataset were manually annotated on the same 247 images as for the previous lung segmentation, and these masks were used to train a U-Net model to segment the spine and use the segmented spine boundary to divide the image into left and right regions. In addition to ROI segmentation and image intensity scaling, normalization was performed to bring all intensities to the same range and enable faster training of the CNN.

#### Step C: Train/test split and data balancing methods

Given a dataset of images, every model generated a prediction output for each image and lung side, which denoted its probability of being classified into one of four classes defined based on degree of lung opacity. The exact quantitative definition of each class is as follows, with percentage values indicating the extent of opacity in the lung: 0 (clear), 1 (1–33%), 2 (34–66%), and 3 (67–100%), examples of which can be seen in Fig. 1.

Initially, an Out Of the Box (OOB) test set of 286 images was created for the purpose of annotation from an additional experienced radiologist and validation of the final model. This OOB test set was not used during the main pipeline for model creation. The remaining patient data was further split into train/test/validation using the ratio of 80, 10, and 10% of patients within the respective splits. Further, to eliminate risk of data leakage between the different splits, we ensured that images from each patient were contained in only one of the above splits. These splits were repeated to create five train/test/validation folds to enable five-fold cross-validation for evaluation, as described in later sections.

The fact that data is not evenly distributed across the four opacity classes within each fold must be addressed, particularly for the training datasets prior to model training. To overcome this issue, three different sampling strategies were explored: (a) random under-sampling (in this strategy, the larger classes were randomly under sampled to reach comparable sample sizes with respect to the smaller classes), (b) double-stage sampling (in this scheme, a two-stage method was developed – with the balanced, randomly undersampled data used for the first stage of fine-tuning of the fully connected network weights and with the entire unbalanced dataset used for the second stage of fine tuning in which the weights of some CNN layers were also changed), and (c) random over-sampling (additional samples of the smaller and larger classes are generated via data augmentation [rotation, scaling, translation] and these samples are resampled randomly across classes such that the final dataset used for training is balanced).

#### Step D: Transfer learning using a multi-level approach

Transfer learning is the concept of transferring knowledge across different but related sources domains, such that knowledge built in one domain is generalizable to a second domain (Zhuang et al., 2019). In our pipeline, we performed transfer learning with fine-tuning of the convolutional layers of the network, along with fine-tuning of the fully connected layers. Performing fine-tuning of the convolutional layers of the network was possible since a large training dataset was available, even after class-balancing methods were applied.

To create a stable initialization for the fine-tuning of the convolutional layers, we adopted a multi-level method (Fig. 2B): (a) first, fine-tune the fully connected layers alone, and (b) second, unfreeze ‘N’ previous convolutional layers and fine-tune them as well. The hyperparameter ‘N’, denoting the number of previous convolutional layers that were fine-tuned, was set to T-5, where T = total number of convolutional layers in the CNN. We performed this multi-level approach using an initial phase of pre-training for a maximum of 20 epochs, followed by fine-tuning for a maximum of 20 epochs (Fig. 2B). Automated learning rate reduction was implemented with patience values of seven epochs, implying that if the validation loss does not decrease for these number of epochs, the learning rate will be reduced. Similarly, an early stopping scheme was also used with a patience value of 10 epochs, such that the model with the lowest validation loss will be selected and saved. The batch size was set to 32, and an ADAM optimizer was used. Since we used a two-stage process for transfer learning (pre-training plus fine-tuning), a learning rate of 0.001 was used for the first stage of the process and 0.0001 for the second stage. Images were down sampled to 224 × 224 prior to going through the network, and all images were scaled between 0 and 1.

#### Step E: Model combinations evaluation

In the first stage of model analysis, combinations of the three image segmentation schemes, three data balancing schemes, and three network architectures were generated (as previously outlined). These combinations resulted in 27 models per lung side and a total of 54 models. Each model was trained, validated, and tested using the first fold of the five folds of data generated above. Each model generated was further evaluated based on precision, recall, F1-score, and macro-averaged mean absolute error (MAE), with all metrics averaged over left and right lungs. The application of macro-averaged MAE to evaluate problems concerning ordinal classification in machine learning in general and medical imaging in particular have been documented widely in literature (Dembczyński et al., 2008; Durán-Rosal et al., 2021). The MAE computes the mean deviation of the predicted class from the true class, thereby penalizing classification errors based on the ordinal scale. In our case, we apply the macro-averaged MAE to also account for class imbalance while penalizing for error. The top five model schemes based on MAE score were retained for further processing in the next stage. In addition to MAE, other metrics such as precision, recall, and F1-score were computed, all of which are defined below for each of the 4 classes.

Consider an example with ‘K’ classes, and the number of samples per class denoted by *n*_*c*_.

The MAE score for a class with the actual opacity score ‘c’ is calculated over all samples *n*_*c*_ belonging to class ‘c’ as:

*MAE*_*c*_ = $\frac{\sum_{i=1}^{n_c}{y}_i-\overline{y_i}}{n_c}$, where $\overline{y_i}$ is the predicted opacity score, and *y*_*i*_ is the actual opacity score.

Further, the overall macro-averaged MAE = $\frac{\sum_{c=1}^K MA{E}_c}{Total\# of\kern0.17em classes\;K\;}$.

The precision, recall, and F1-score are all computed as weighted averages of the per-class metrics.

The precision for class ‘c’ is defined as: Precision = ${P}_c=\frac{True\kern0.17em positive}{True\kern0.17em positive+ False\kern0.17em positive}$;

From this, overall precision is computed as: ${P}_{overall}=\frac{P_c\times {n}_c}{\sum_{c=1}^K{n}_c}$.

The recall for class ‘c’ is defined as: *R*_*c*_ = $\frac{True\kern0.17em positive}{True\kern0.17em positive+ False\kern0.17em negative}$;

Based on this, the overall recall across all classes is computed as: *R*_*overall*_ = $\frac{R_c\times {n}_c}{\sum_{c=1}^K{n}_c}$.

From the above computations of precision and recall, the F1-score per class is calculated as:

*F*1_*c*_ = $\frac{2\times {P}_c\times {R}_c}{P_c+{R}_c}$, where *P*_*c*_ and *R*_*c*_ refer to the precision and recall for class ‘c’.

Finally, the overall F1-score is computed as: *F*1_*overall*_ = $\frac{F{1}_c\times {n}_c}{\sum_{c=1}^K{n}_{c.}}$

#### Step F: 5-fold cross validation of top five models

In the second stage of model analysis, the top five models from the first stage are further retrained and evaluated across all the five folds of data generated. All metrics analyzed previously, along with a new metric based on heatmap concordance, were computed across all five folds. The addition of the heatmap concordance score (HCS) ensured that interpretability and clinical relevance of chosen features were quantified and played a key role in choice of best model.

#### Step G: Stage 3 – final model training and testing

In the final stage of model analysis, the best model from Stage 2 is trained on an entirely new train-test split of the data. In this stage, the resulting model predictions are also evaluated against newly annotated scores assigned by an expert radiologist, referred to as the OOBTR or Out Of Box Test Reader throughout the manuscript. The role of the OOBTR is to provide a second reader score at the final analysis stage, to enable inter-reader comparison of annotated scores. Using both reader scores as reference, model-reader agreement can be compared with agreement between readers. Both heatmap scores and MAE are evaluated for model prediction versus annotations by the Original Reader at time of X-ray acquisition (OR), Out Of Box Test Reader (OOBTR) during the time of testing, and the heatmaps are visualized for different scenarios in a matrix form.

#### Step H: Heatmap generation

Saliency maps describing the importance of image regions in the decision-making process of the classifier were generated for each class. These maps were generated for each output class using the gradient-weighted class activation mapping (Grad-CAM) visualization method by global pooling of gradients that are flowing backward from the last convolutional layer of the CNN (Rajaraman et al., 2020; Selvaraju et al., 2020).

**Fig. 3:**
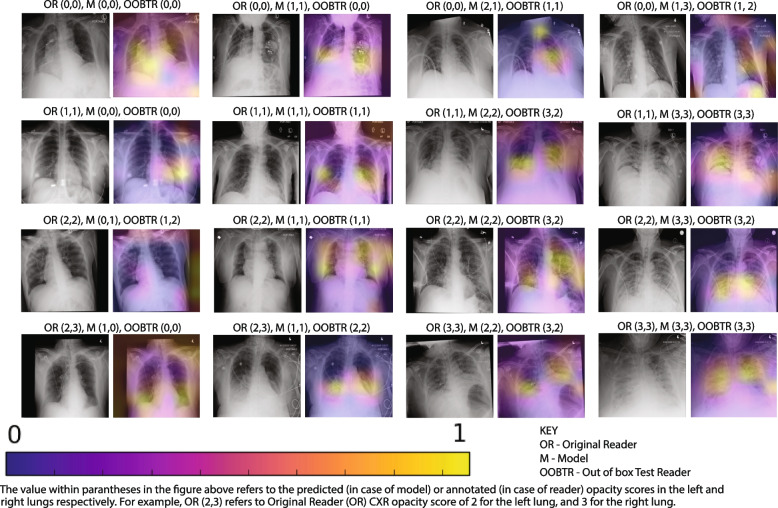
Examples of generated heatmaps and classifications from the model, the OR, and the OOBTR. The value within the parantheses in the figure refers to the predicted (in case of model) or annotated (in case of reader) opacity scores for the left and right lungs respectively. For example, OR (2,3) indicates an Original Reader (OR) CXR opacity score of 2 for the left lung, and 3 for the right lung

Following the generation of per-class saliency maps, heatmaps describing network ‘attention’ were generated by weighted averaging of the saliency maps of all classes with weights determined by the network’s output probabilities for each class. The resulting heatmaps indicate salient regions that the network is ‘looking at’ during its classification process (Fig. 3). Finally, heatmaps for the left-lung and right-lung specific classifiers were averaged to obtain an averaged heatmap for the whole lung.

##### Heatmap score

To compute the novel HCS, we compute the ratio of all non-zero heatmap pixels within the lung boundary region to all non-zero heatmap pixels in the total image. The computation of the lung boundary region is done via the U-Net segmentation algorithm described in the earlier section. The averaged heatmap score from left and right lungs was multiplied with the mask to generate a measure of overlap between heatmap salience and the lung boundary.

$$\frac{Lung\kern0.17em Mask\times \left( heatma{p}_{left}+ heatma{p}_{right}\right)/2}{\left( heatma{p}_{left}+ heatma{p}_{right}\right)/2}$$

The final methodology used to assess model performance is a combination of the macro-averaged MAE score and the macro-averaged heatmap score, with both assessed jointly in a balanced manner.

#### Step I: Performance analysis across distinct patient populations

To observe and compare model accuracy and performance across distinct patient populations, the model that was trained on the master dataset was tested on patient subgroups and performance metrics were noted. The distinct patient subgroups were generated based on demographic data, and the following categories of patients were evaluated: (a) COVID-19 status (i.e., COVID-19 positive, COVID-19 negative), (b) race (i.e., Asian, Black, White, Other, Unknown), and (c) sex (i.e., male, female).

## Results

### Patient population

The initial set of 83,197 DICOM images (20,180 patients) were processed. After application of exclusion criteria (Fig. 2) 38,365 images, obtained from 17,418 patients, remained in total. Summary statistics of patient demographics and image statistics for the overall dataset and the train/test/validation and OOB test groups are included in Tables 1 and 2. Patient-wise statistics, shown in Table 1, include sex (male/female), patient status (inpatient, outpatient, and ED), COVID-19 status (positive/negative), and race. Image-wise statistics, shown in Table 2, include X-ray view (anterior-posterior/posterior-anterior) and CXR opacities. Both patient and image statistics maintain similar distributions/values across the different data splits.

**Table 1:** Demographics distributions for the corpus under study in terms of patients

Patients	All Data	Train	Test	Validation	OOB Test
Patient Count N (%)	17,418 (100)	13,808 (79.27)	1726 (9.99)	1725 (9.90)	159 (0.91)
Image Count N (%)	38,365 (100)	30,586 (79.72)	3660 (9.53)	3833 (9.99)	286 (0.74)
Age Mean (stdev)	60.88 (16.98)	60.89 (16.98)	60.81 (17.30)	60.78 (16.70)	62.44 (15.77)
**Sex**					
Male (%)	8983 (51.57)	7123 (51.58)	903 (52.31)	873 (50.60)	84 (52.83)
Female (%)	8435 (48.42)	6685 (48.41)	823 (47.68)	852 (49.39)	75 (47.16)
**Patient Status**					
IP (%)	10,246 (58.82)	8096 (58.63)	1003 (58.11)	1043 (60.46)	104 (65.4)
OP (%)	197 (1.13)	158 (1.14)	18 (1.04)	17 (0.98)	4 (2.51)
ED (%)	6975 (40.04)	5554 (40.22)	705 (40.84)	665 (38.55)	51 (32.07)
**COVID-19 Status**					
Positive (%)	6692 (38.42)	5259 (38.08)	672 (38.93)	698 (40.46)	63 (39.62)
Negative (%)	10,725 (61.57)	8548 (61.90)	1054 (61.06)	1027 (59.53)	96 (60.37)
Unknown (%)	1 (0.005)	1 (0.007)	0 (0.00)	0 (0.00)	0 (0.00)
**Race**					
Black (%)	3153 (18.10)	2476 (17.93)	322 (18.65)	329 (19.07)	26 (16.35)
White (%)	7942 (45.59)	6264 (45.36)	802 (46.46)	801 (46.43)	75 (47.16)
Asian (%)	1349 (7.74)	1074 (7.77)	129 (7.47)	136 (7.88)	10 (6.28)
Other (%)	4120 (23.65)	3304 (23.92)	391 (22.65)	380 (22.02)	45 (28.30)
Unknown (%)	854 (4.90)	690 (4.99)	82 (4.75)	79 (4.57)	3 (1.88)

Patient-wise demographics for age, sex, patient status, COVID-19 status, and race.

**Table 2:** Demographics distributions for the corpus under study in terms of images

Images	AllData	Train	Test	Validation	OOB Test
Image Count N (%)	38,365 (100)	30,586 (79.72)	3660 (9.53)	3832 (9.99)	286 (0.74)
**Xray view**					
PA (%)	456 (1.18)	374 (1.22)	39 (1.06)	40 (1.04)	3 (1.04)
AP (%)	36,596 (95.38)	29,165 (95.35)	3491 (95.38)	3665 (95.61)	275 (96.15)
Unknown (%)	1313 (3.42)	1047 (3.42)	130 (3.55)	128 (3.33)	8 (2.79)
**CXR opacity - left**					
None	12,488 (32.55)	9983 (32.63)	1234 (33.71)	1174 (30.62)	97 (33.91)
Mild	12,381 (32.27)	9798 (32.03)	1189 (32.48)	1300 (33.91)	94 (32.86)
moderate	8310 (21.66)	6625 (21.66)	760 (20.76)	862 (22.48)	63 (22.02)
severe	5185 (13.51)	4180 (13.66)	477 (13.03)	496 (12.94)	32 (11.18)
**CXR opacity - right**					
None	12,938 (33.72)	10,237 (33.46)	1297 (35.43)	1311 (34.21)	93 (32.51)
mild	11,382 (29.66)	9080 (29.68)	1051 (28.71)	1171 (30.55)	80 (27.97)
moderate	8459 (22.04)	6771 (22.13)	792 (21.63)	821 (21.42)	75 (26.22)
severe	5585 (14.55)	4498 (14.70)	520 (14.20)	529 (13.80)	38 (13.28)

Image-wise demographics for CXR view and opacity degree.

### Model training and testing

After training all 27 models using 30,586 images with test and validation sets of 3660 and 3833 images, we measured their performance using the precision, recall, F1, and macro-averaged MAE metrics (Table 3). All models were ranked according to MAE score and the top five models were chosen to be further evaluated using a five-fold cross-validation technique. One may observe that models with CheXNet-121, ResNet-50, and VGG-16 architectures along with the data preprocessing of no-ROI segmentation, or spine-based ROI segmentation, and sampling strategies of double-stage or under-sampling were among the top-performing models. These models, which were in the final top five retained for further processing, had an MAE score of 0.40–0.41 and F1 score ranging from 62 to 64%. The entire dataset of models had F1 scores ranging from 38 to 64%, indicating the wide variability based on model parameters. Models with data balancing strategies involving over-sampling and data preprocessing comprised of lung-based ROI segmentation did not perform as well as the other models, as per the macro-averaged MAE.

**Table 3:** Comparison of all models for Fold 1 and choosing top five models by MAE

Model Architecture	Data Balancing Strategy	Segmentation Type	Precision	Recall	F1	MAE
**CheXNet-121**	**UNDER**	**NONE**	**65.18**	**64.90**	**64.41**	**0.3953**
**CheXNet-121**	**DOUBLE**	**NONE**	**64.88**	**65.02**	**64.62**	**0.4023**
**ResNet-50**	**UNDER**	**NONE**	**63.62**	**63.46**	**62.82**	**0.4097**
**ResNet-50**	**DOUBLE**	**SPINE**	**63.85**	**64.24**	**63.91**	**0.4144**
**VGG-16**	**DOUBLE**	**SPINE**	**62.89**	**62.54**	**62.60**	**0.4151**
VGG-16	UNDER	NONE	62.60	62.32	62.24	0.4161
CheXNet-121	DOUBLE	SPINE	63.37	63.62	63.20	0.4193
VGG-16	DOUBLE	NONE	63.45	62.76	62.36	0.4210
CheXNet-121	UNDER	SPINE	63.55	63.62	63.25	0.4214
ResNet-50	DOUBLE	NONE	63.02	63.75	62.77	0.4238
ResNet-50	UNDER	SPINE	63.79	62.72	62.60	0.4279
VGG-16	OVER	LUNG	60.43	61.37	61.06	0.4320
VGG-16	OVER	NONE	60.81	60.98	60.11	0.4371
CheXNet-121	OVER	NONE	61.56	61.76	60.85	0.4392
ResNet-50	DOUBLE	LUNG	62.54	62.22	62.14	0.4412
VGG-16	UNDER	SPINE	62.32	62.43	61.56	0.4439
ResNet-50	UNDER	LUNG	61.31	61.38	60.93	0.4454
CheXNet-121	OVER	SPINE	61.13	61.24	60.80	0.4489
ResNet-50	OVER	LUNG	59.63	60.58	59.64	0.4600
CheXNet-121	OVER	LUNG	58.94	59.23	58.39	0.4613
VGG-16	UNDER	LUNG	60.95	60.17	60.02	0.4612
VGG-16	DOUBLE	LUNG	61.01	61.07	60.73	0.4635
ResNet-50	OVER	SPINE	60.39	61.20	60.37	0.4665
CheXNet-121	UNDER	LUNG	60.55	60.72	60.40	0.4669
ResNet-50	OVER	NONE	59.54	57.31	56.07	0.4864
CheXNet-121	DOUBLE	LUNG	59.58	60.60	59.68	0.4973
VGG-16	OVER	SPINE	35.79	46.46	38.04	0.9625

### Selection of best model from top five models

Five-fold cross validation was performed on the top five models from Table 3 to provide a more thorough and rigorous evaluation of them and to establish model robustness and reliability. Table 4 shows the metrics defined above along with mean and standard deviation ranges over the five-fold cross-validation experiments. MAE values of the top five models ranged between 0.38–0.45, and F1 scores ranged from 61 to 65%. While MAE values of the top three models were very close (with an MAE range of 0.39–0.405, within .01 of MAE score), the macro-averaged heatmap concordance score of the fine-tuned ResNet-50 models were much superior (0.18 versus 0.13). Upon further examination, we observed that for the CheXNet-121 model, several moderate and severe opacity images, as per the original radiologists annotation, had heatmaps with low clinical concordance, as the salient regions as delineated by the heatmap were partly outside the lung boundary regions. Comparisons of these models for binary outcomes (presence or absence of opacities, none/mild or medium severe opacities, severe or not severe opacities) were also calculated and the three main performance metrics (precision, recall and F1) are listed in Table 5.

**Table 4:** Robustness comparison of final models across all five folds and choice of best model

Model Architecture	Data Balancing Strategy	Segmentation Strategy	Precision Mean (stdev)	Recall Mean (stdev)	F1 Mean (stdev)	MAE Mean (stdev)	MA HCS Mean
CheXNet-121	DOUBLE	NONE	65.89 (1.28)	66.30 (1.13)	65.82 (1.18)	0.3944 (0.0147)	0.1481
CheXNet-121	UNDER	NONE	65.36 (1.26)	65.47 (1.15)	65.06 (1.32)	0.3930 (0.0126)	0.1352
ResNet-50	UNDER	NONE	64.88 (1.10)	64.81 (0.82)	64.33 (0.85)	0.4099 (0.0147)	0.1830
ResNet-50	DOUBLE	SPINE	64.33 (1.21)	64.37 (0.99)	63.90 (1.19)	0.4149 (0.0157)	0.2057
VGG-16	DOUBLE	SPINE	62.02 (1.31)	61.18 (1.27)	61.44 (1.27)	0.4325 (0.0182)	0.1631

**Table 5:** Comparison of final models across all five folds for binary models

Model Architecture	Data Balancing Strategy	Segmentation Strategy	Precision Mean (Stdev)	Recall Mean (Stdev)	F1 Mean (Stdev)
(A) None versus mild/medium/severe					
CheXNet-121	DOUBLE	NONE	93.47 (0.92)	88.07 (0.78)	90.69 (0.7)
CheXNet-121	UNDER	NONE	92.70 (0.93)	88.76 (1.29)	90.68 (0.78)
ResNet-50	UNDER	NONE	92.61 (1.7)	88.08 (1.86)	90.25 (0.68)
ResNet-50	DOUBLE	SPINE	92.05 (2.03)	88.18 (2.15)	90.03 (0.63)
VGG-16	DOUBLE	SPINE	79.63 (1.85)	79.63 (2.29)	81.46 (0.82)
(B) None/mild versus medium/severe					
CheXNet-121	DOUBLE	NONE	80.74 (1.38)	81.91 (2.51)	81.29 (1.44)
CheXNet-121	UNDER	NONE	78.70 (1.83)	84.81 (1.6)	81.62 (1.1)
ResNet-50	UNDER	NONE	80.06 (2.42)	81.24 (4.72)	80.46 (1.66)
ResNet-50	DOUBLE	SPINE	79.12 (1.7)	81.65 (4.09)	80.24 (1.41)
VGG-16	DOUBLE	SPINE	79.63 (2.14)	83.38 (4.27)	79.64 (2.07)
(C) None/mild/medium versus severe					
CheXNet-121	DOUBLE	NONE	62.91 (3.34)	59.32 (4.83)	60.91 (2.91)
CheXNet-121	UNDER	NONE	58.96 (4.09)	64.97 (3.24)	61.61 (2.27)
ResNet-50	UNDER	NONE	64.43 (3.8)	54.61 (8.26)	58.42 (4.16)
ResNet-50	DOUBLE	SPINE	62.05 (3.81)	57.43 (6.92)	59.24 (3.85)

Given these results, upon examining both the F1 scores as well as the HCS values, the best model overall was the fine tuned ResNet-50 model, with undersampling as the data balancing scheme, and no ROI based segmentation as the preprocessing step. F1-score of this model was 64.33%, MAE was 40.99 and HCS score was 0.18.

### Evaluation of top model compared to expert radiologist reader

Following model comparison based on the deep learning framework described above, predictions of model opacity based on the top model were obtained for the OOB (Out of Box) test set. Multiple evaluation metrics such as the MAE, F1-score, precision, and recall were applied to determine the model performance (Tables 6 and 7). The MAE in particular evaluates the difference between expected and predicted values, and was crucial in determining the model performance given the ordinal nature of the data. Other metrics include: Precision, which computes the percentage of predictions that an image had opacity score ‘A’ that were correct; Recall, which computes the percentage of correctly recognizing an image with opacity ‘A’; and F1-score, which gives a single score that balances precision and recall via calculating the harmonic mean of these values.

**Table 6:** Output comparison between final model, OR (Original Reader), and OOBTR (Out Of Box Test Reader). Model output compared with OOBTR and OR using multiple evaluation metrics for the multiclass classification problem

	O.O.B.T.R.- O.R.	Model- O.R.	Model- O.O.B.T.R.
**MAE**	0.4560	0.3305	0.4316
**Precision**	61.63	68.31	71.18
**Recall**	62.05	67.12	68.70
**F1**	61.38	66.99	69.05
**R-squared**	0.55	0.614	0.6769

**Table 7:** Output comparison between final model, OR (Original Reader), and OOBTR (Out Of Box Test Reader). Model output compared with OOBTR and OR using multiple evaluation metrics for multiple binary classifications with binary output classes derived from the original multiclass output values

	O.O.B.T.R.- O.R.	Model- O.R.	Model- O.O.B.T.R.
**Absence or Presence of Opacity**			
**Precision**	92.50	88.64	88.89
**Recall**	89.53	91.19	93.62
**F1**	90.95	89.89	91.12
R-squared	0.45	0.42	0.49
**No Opacity, Mild Opacity VS. Medium & Severe Opacity**			
**Precision**	77.53	81.31	82.57
**Recall**	75.08	84.23	84.08
**F1**	76.25	82.51	83.01
**R-squared**	0.21	0.44	0.46
**Not Severe or Severe**			
**Precision**	46.64	55.27	71.56
**Recall**	62.57	80.64	69.53
**F1**	53.45	65.45	70.32
**R-squared**	0.06	0.02	0.28

The following combinations of predictions are evaluated using the metrics mentioned above: (a) OOBTR (Out of Box test Reader) score vs. the OR (Original Reader) score, (b) Model score vs. the OR (original reader) score, and (c) Model score vs. the OOBTR (out of box test reader) score.

In comparing classification outputs between our final model, the OR (Original Reader), and the OOBTR (Out Of Box Test Reader), we found that that the current model has MAE of 0.4316 when its output was compared with the OOBTR and an MAE of 0.3305 when its output was compared with the OR, whereas the OOBTR-OR comparison had an MAE of 0.456. Other evaluation metrics, including the F1-score, precision, recall, and R-squared errors, were also reported. It was observed that the Model predictions compared with the OR and OOBTR scores has a higher recall score (67.12 and 68.70), higher precision (68.31 and 71.18), and higher F1-score (66.99 and 69.05) in contrast with the recall, precision, and F1-score from the OR-OOBTR score comparisons (62.05, 61.63, and 61.38 respectively). These results demonstrate that the model’s predicted scores were closer to the OR and OOBTR scores as opposed to the OR and OOBTR scores, which were further apart upon comparison.

Multiple binary comparisons were also shown in terms of these evaluation metrics (prediction of no opacity versus mild, medium, and severe opacity; prediction of no opacity and mild opacity versus medium and severe opacity; and prediction of no opacity, mild, and medium opacity versus severe opacity). Across all categories in the multiple binary comparisons, excluding precision and f1 scores in the presence or absence of opacity comparison, it was observed that Model-OR and Model-OOBTR performance metrics had consistently higher values than the OR-OOBTR metrics. This is particularly apparent in the case of the severe vs. not severe binary comparison, in which the Model-OR and Model-OOBTR F1-scores (65.45 and 70.32 respectively) where over 10 points higher than the OR-OOBTR F1-score (53.45).

Finally, visual inspection of the resulting heatmaps in different levels of agreement between predicted and annotated labels reveals that, in all cases of complete agreement between the model and the expert radiologist reader, the ROI of the model focuses on clinically relevant areas of CXRs (Fig. 3).

### Performance analysis of best model across population groups based on race, sex, and COVID-19 status

Performance metrics were also computed across different population groups, based on race, sex and covid status (Table 8). A comparison of macro-averaged MAE scores across different racial groups shows that average MAE scores span a range, varying from 0.3971 (Asian) to 0.4626 (Black). A similar comparison of MAE across patients grouped based on sex showed that MAE is 0.4129 for the female group and 0.4295 for the male group. The MAE of patients grouped based on COVID-19 status showed variability from 0.4216 for the COVID-19–positive group to 0.4584 for the COVID-19–negative group. F1-scores across all groups showed variability from 61.58 to 67.44.

**Table 8:** Performance analysis of best model

Population	N	Avg MA- MAE	Lt MA-MAE	Rt MA- MAE	Avg F1	Lt F1	Rt F1	Avg Pr	Lt Pr	Rt Pr	Avg Rec	Lt Rec	Rt Rec
All Patients	2839		0.4769	0.3855		62.11	65.21		62.24	66.22		63.08	65.41
**RACE**													
Asian	184	0.3971	0.4536	0.3406	64.25	62.72	65.78	65.56	62.57	68.54	65.76	64.67	66.84
Black	453	0.4626	0.5408	0.3844	64.34	60.98	67.69	65.54	62.75	68.33	65.45	62.69	68.21
White	1346	0.438	0.4736	0.4025	64.08	63.34	64.82	64.36	63.26	65.45	64.37	63.96	64.78
Other	739	0.4196	0.4579	0.3813	62.00	60.37	63.64	63.39	60.83	65.95	62..65	61.43	63.87
Unknown	117	0.4039	0.4510	0.3569	64.45	60.73	68.16	65.59	61.45	69.73	65.81	62.39	69.23
**SEX**													
Male	1625	0.4295	0.4937	0.3652	63.16	60.07	66.25	64.03	60.63	67.43	63.75	61.16	66.33
Female	1214	0.4129	0.4482	0.3775	66.94	65.68	68.20	67.56	65.64	69.48	67.58	66.63	68.53
**COVID**													
COVID +ve	1513	0.4216	0.4783	0.3649	61.58	55.40	67.76	62.44	69.64	66.32	62.12	55.71	68.53
COVID -ve	1326	0.4584	0.5262	0.3907	67.44	69.46	65.43	67.98	56.29	68.59	67.87	70.58	65.16

The final model was comprised of a fine-tuned ResNet-50 architecture with no ROI segmentation and an undersampling scheme. The performance analysis is performed across race, sex and COVID-19 status. Abbreviations used in the table include Pr = Precision, Rec = Recall, F1 = F1 score, and MA-MAE = Macro-averaged Mean Absolute Error

## Discussion

We developed a robust deep transfer learning framework that was adapted to estimate CXR lung opacity using transfer learning and then validated it against annotations by expert radiologists. Our framework provides a comprehensive pipeline to perform machine learning analysis on radiographic images.

Although several studies have built models to detect pneumonia, few studies have annotated lung images beyond present/absent labels and do not include information regarding degrees of opacity present (Roberts et al., 2021). The degree of lung opacities on CXRs have been shown to predict patient outcomes (Au-Yong et al., 2022; Balbi et al., 2021; Voigt et al., 2022) and, therefore, the ability to make this distinction accurately may prove vital in future studies. Pulmonary opacity, which represents air/gas in the alveolar space replaced by a process such as fluids or cells, has been closely related to both pulmonary disease severity and prognosis (Au-Yong et al., 2022; Balbi et al., 2021; Voigt et al., 2022). Recent studies have developed algorithms to compute opacity-related scores using deep learning frameworks (Cohen et al., 2020; Li et al., 2020), though they have been limited by a low number of COVID-19 training or test images (up to 300 and 200 images, respectively). Data augmentation (Mutasa et al., 2020), regularization with dropout, or weight decay (Yamashita et al., 2018) and transfer learning are commonly used strategies to reduce risk of overfitting when using relatively small numbers of training and testing data. However, to avoid variable generalization of such models (Yamashita et al., 2018), larger numbers of images from a diverse patient population, similar to what we showcase in this study, not only help with overfitting but reduce the possibility of reflecting or enhancing biases (Seyyed-Kalantari et al., 2021).

The choice of convolutional neural network architectures in radiology machine learning studies is not usually accompanied by systematic comparisons of the performance of other model architectures on similar use cases or similar data. Over 50% of COVID-19 diagnostic and prognostic radiology machine learning models use the ResNet-18, ResNet-50, or DenseNet-121 architectures (of which CheXNet is an example), with a few papers opting for VGG-16 or VGG-19, EfficientNet, InceptionNet, custom architectures, or hand-engineered features instead (Roberts et al., 2021). ResNet and DenseNet architectures generally showed superior performance over other architectures, though this comparison is not comprehensive since these architectures were not tested against a single standardized dataset. Moreover, similarly important to the choice of the network architecture are strategies on handling imbalanced datasets and use of segmentation steps prior to training the networks (Johnson & Khoshgoftaar, 2019; Malhotra et al., 2022). In our work, we seek to conduct a thorough comparison of these architectures, data balancing schemes for handling bias, and image preprocessing strategies for ROI extraction using a single dataset with extensive cross-validation. Conducting this exhaustive comparison in a computationally effective manner can remove some heuristic aspects of selecting network architectures and other preprocessing and training parameters and offers a more rigorous and efficient approach to develop such models.

Transfer learning has been an invaluable tool in medical imaging studies that use machine learning methods and, specifically, CNNs (Alzubaidi et al., 2021). Transfer learning across multiple domains refers to using a pretrained network trained on a different dataset, and then using this as a starting point for further training or fine tuning with respect to the target task, an approach that is very popular in the medical imaging field (Cheplygina et al., 2019). While our modeling scheme leverages pretrained weights from ResNet-50, CheXNet-121, and VGG-16 models, instead of only training the fully connected network (FCN), we also train the last ‘N’ layers (five layers for ResNet-50 and VGG-16 and 15 for CheXNet-121) of our CNN. Our results demonstrate that this two-stage finetuning scheme, with the first stage consisting of fine tuning the FCN and the second stage consisting of fine tuning the CNN, produces superior results, with an increased F1 score of at least 3–5%, which can be empirically validated.

An important aspect of developing and validating machine learning algorithms that use medical images is the correct choice of performance metrics (Hicks et al., 2022). While binary classifications can be evaluated using commonly used metrics such as precision/recall/F1 score, these are not as effective in cases where ordinal labels indicating progressive lung opacities are seen due to pneumonia; new strategies need to be evolved to address this use case. In our work, we apply the macro-averaged MAE, designed specifically to evaluate models with multiple ordinal labels as output while also accounting for class imbalance in the test sample. Further, we account for whether regions that are salient to the model’s decision overlap with clinically relevant regions using the HCS. To create a single quantitative index of model performance, our final choice of model is decided based on a combination of both the above scores.

Prior studies reported that F1 scores of comparisons of COVID-19 versus non–COVID-19 lung images ranged from 89 to 97% when evaluated on test sets ranging from just 100 test images to 3000 (Roberts et al., 2021). Meanwhile, in a study examining the classification accuracy of models for determining presence/absence of pneumonia prior to COVID-19, an F1 score of 76.80% was reported based on a test set of 420 images (Rajpurkar et al., 2017). In our study, our methodology produces an F1 score of 91% when testing for presence or absence of opacity (corresponding to presence/absence of pneumonia). In our work, the number of test images was higher than all prior studies mentioned (2839 test images plus 286 for out-of-box testing, resulting in 3125 images in total). Further, a study aimed at determining the geographic extent of opacities in CXR’s of COVID-19 patients showed an ${}^{R^2}$ score of 0.67, which is comparable to the ${}^{R^2}$ score of our best model compared with expert radiologist at time of testing (0.6769) and upon receiving the Xray (0.614). (Cohen et al., 2020).

The algorithm has the ability to detect lung opacities as well as grade the severity of those opacities. With validation, the ability to detect opacities is helpful for clinicians to ensure lung abnormalities are not missed and to prioritize chest x-rays that need further evaluation. Further, clinical correlation in the setting of these opacities can change patient care. The ability to grade degree of opacity needs further evaluation to understand its ability to predict key patient outcomes and how it can be best used in clinical care.

Our study has some limitations, especially pertaining to multiple disease processes. Infections, edema, neoplastic processes, and inflammatory conditions can all produce lung opacities that clinicians use to both determine the extent of the underlying disease and monitor for disease course. We do not distinguish between these various disease processes.

A limitation of the current framework is that the 54 models being tested in the first stage are not tested using multiple cross-validation folds. Due to limitations of available computational power, testing in this manner would be beyond the scope of this paper. Also due to computational limitations, we chose to use pretrained models for classification along with transfer learning rather than training from scratch. However, comparison of the pretrained models with a model created from scratch might also be informative and provide increased accuracy.

The degree of opacity on CXRs was determined by radiologists in real time, allowing us to quantify a large volume of images. However, the fact that this was done under the demands of clinical care during the initial wave of the COVID-19 pandemic may have limited accuracy. To account for this, 286 CXR images were reviewed by an expert cardiothoracic radiologist in a research setting. Further, CXRs are less accurate than computed tomography for determining the presence or absence of lung opacities, though they are the most ubiquitous imaging test for diagnosing respiratory diseases worldwide and, therefore, they need to have their diagnostic capabilities improved (Mettler Jr. et al., 2009). Finally, this study was conducted within one, albeit large, health system; this potentially limited the generalizability of our ML algorithm.

## Conclusion

In conclusion, we developed a robust deep learning framework to analyze radiographic images and adapted it to estimate CXR opacity. The framework tests all combinations of several neural network architectures, segmentation, and data balancing strategies in a rigorous and computationally efficient manner and selects optimal configurations based on the dataset and use case. When applied to 38,365 CXR, it performed as accurately as experienced radiologists, regardless of COVID-19 status or race of the patient. Accurate machine learning characterization of lung opacity on CXR could have numerous potential clinical applications. Lung opacity seen in CXRs has been shown to predict patient outcomes in entities such as COVID-19 and lung edema (Au-Yong et al., 2022; Voigt et al., 2022). Further work is needed to determine whether our CXR ML algorithm can be used to predict patient outcomes.

## Data Availability

The datasets used and/or analysed during the current study are available from the corresponding author on reasonable request. Code can be found at https://github.com/Avardhannorthwell/ALOE_CXR.

## Abbreviations

CNN: Convolutional Neural Network
CXR: Chest Xray or Chest Radiograph
DenseNet: Dense Network
ED: Emergency Department
FCN: Fully Connected Network
GRADCAM: GRADient weighted Class Activation Mapping
HCS: Heatmap Concordance Score
IP: Inpatient
JSRT: Japanese Society of Radiological Technology
MA: Macro Averaged
MAE: Mean Absolute Error
OOB Test: Out Of Box Test
OOBTR: Out Of Box Test Reader
OP: Outpatient
OR: Original Reader
PCR: Polymerase Chain Reaction
ResNet: Residual Network
ROI: Region Of Interest
